# Synovial Sarcoma: Malignant Soft Tissue Sarcoma With Benign Clinical Characteristics—A Case Report

**DOI:** 10.1155/crip/9585628

**Published:** 2025-09-01

**Authors:** Jay Lodhia, David Msuya, Joshua Tadayo, Alex Mremi

**Affiliations:** ^1^Department of General Surgery, Kilimanjaro Christian Medical Centre, Moshi, Tanzania; ^2^Faculty of Medicine, KCMC University, Moshi, Tanzania; ^3^Department of Renal & Pancreatic Transplantation, Manchester University NHS Foundation Trust, Manchester, UK; ^4^Department of Pathology, Kilimanjaro Christian Medical Centre, Moshi, Tanzania; ^5^Kilimanjaro Clinical Research Institute, Moshi, Tanzania

**Keywords:** case report, hand, soft tissue neoplasm, synovial sarcoma, Tanzania

## Abstract

Synovial sarcomas are rare malignant soft tissue tumors with significant metastatic potential. Although they can occur in various parts of the body, they are most commonly found on the extremities. These tumors typically develop in children and young adults, making occurrences in individuals over 50 years of age unusual. Due to their slow-growing and nonpainful nature, synovial sarcomas can often be mistaken for benign pathologies. The standard treatment involves complete surgical excision with negative margins, which offers a favorable 5-year prognosis. This case highlights the importance of early recognition and intervention in managing soft tissue sarcomas. In this case report, we present a 57-year-old African male with a 2-year history of gradual, nonpainful swelling on his left hand, diagnosed as synovial sarcoma. The patient was successfully treated with complete surgical excision.

## 1. Introduction

Synovial sarcoma is a rare and aggressive soft tissue malignancy, comprising 5%–10% of all soft tissue sarcomas. The incidence is approximately 0.81 per million in children and 1.42 per million in adults, affecting both genders equally, predominantly in adolescents and young adults [1]. Despite its name, synovial sarcoma does not originate from synovial tissue, and it is characterized by a high potential for metastasis. A hallmark of this malignancy is the presence of a specific translocation between chromosomes X and 18, leading to the formation of various fusion proteins, which are detectable in over 95% of cases, making them a crucial diagnostic marker [2].

Surgical excision remains the cornerstone of synovial sarcoma treatment, with the aim of removing the tumor along with a margin of healthy tissue to reduce the risk of recurrence. While adjuvant radiation and/or chemotherapy may be considered for high-risk patients, the benefits of these treatments are still a subject of ongoing research and debate [3].

According to our literature review, this is the first reported case of synovial sarcoma from Tanzania, with few—if any—cases documented from sub-Saharan Africa. This makes it an important case for publication, as it contributes to extending current knowledge, filling a significant geographic and epidemiological gap, and highlighting the need for further case reporting from such regions. Documenting cases from these areas may also reveal new associations, such as a possible link to HIV infection, and improve understanding of disease presentation and management in different populations.

In this case report, we presented a middle-aged male patient with a 2-year history of a progressively enlarging mass on his left hand, which had not responded to various over-the-counter and herbal medications. The mass was successfully excised in its entirety, with no signs of recurrence observed during follow-up. This case underscores the importance of early diagnosis and surgical intervention in the management of synovial sarcoma to achieve favorable outcomes.

## 2. Case Presentation

A 57-year-old African male, who is HIV-positive and on regular ARV therapy, presented with a gradually increasing swelling on his left hand over the past 2 years. The swelling was primarily located on the palmar aspect between the thumb and index finger. It was associated with mild, dull pain that radiated to the left wrist joint, accompanied by a tingling sensation and a reduced range of motion in the thumb and index finger. There was no history of trauma to the hand, no gross swelling of the left upper limb, and no systemic symptoms such as fever or weight loss.

During the course of his illness, the patient attempted various over-the-counter medications, including topical herbal treatments from traditional healers, but none provided relief. He eventually sought medical attention when the functional impairment of his hand progressed to the point where he was unable to perform some of his daily tasks.

**Figure 1:**
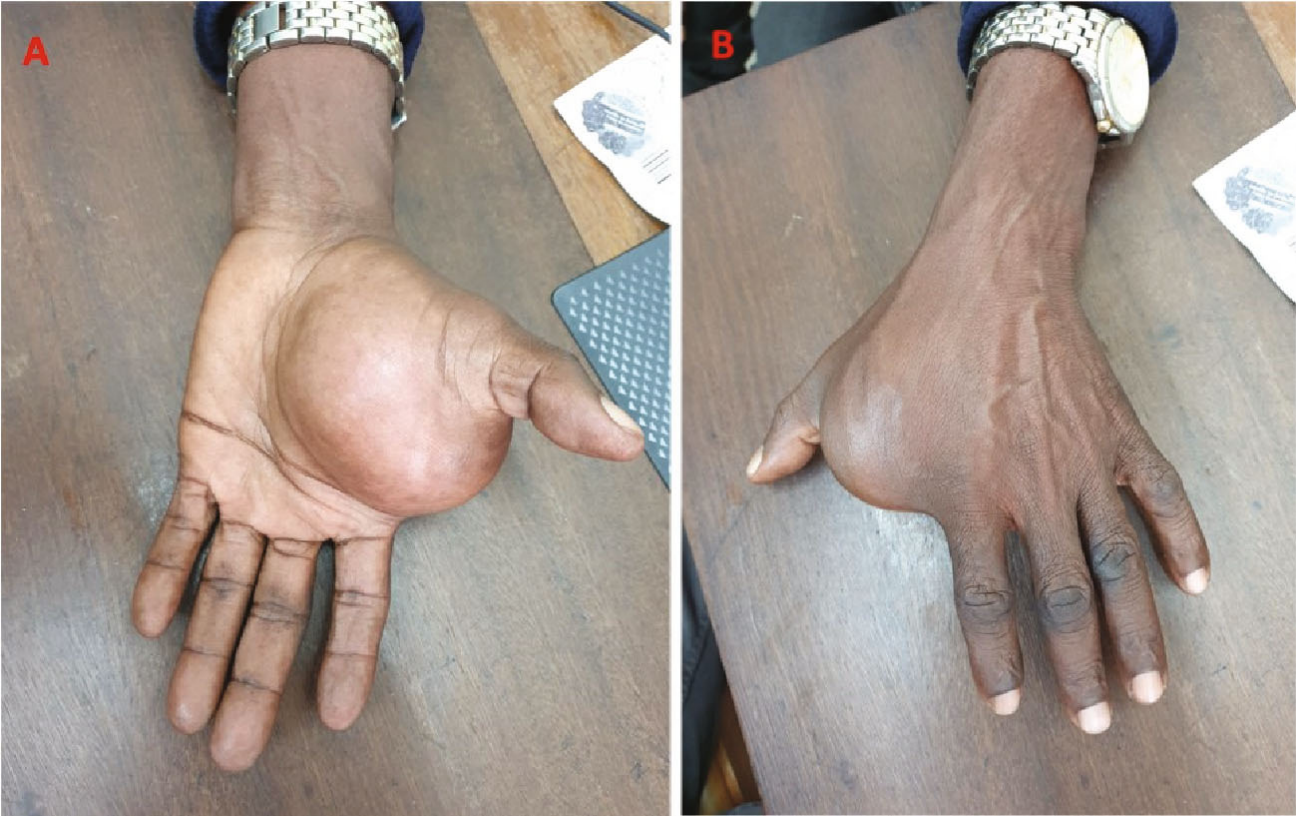
Clinical photograph showing synovial sarcoma between the index finger and thumb: more prominent on the (A) palmer side compared to (B) dorsum.

On examination, the patient appeared well, with no signs of pallor or jaundice and no palpable lymphadenopathy. His vital signs were stable. The left hand exhibited a localized swelling measuring 12 × 10 cm on the palmar aspect, extending to the dorsum of the hand between the thumb and index finger. The skin over the mass was normal, with no ulcerations. The mass had smooth edges, a firm consistency, and was nontender, with no palpable pulsatile sensation (Figure 1).

**Figure 2:**
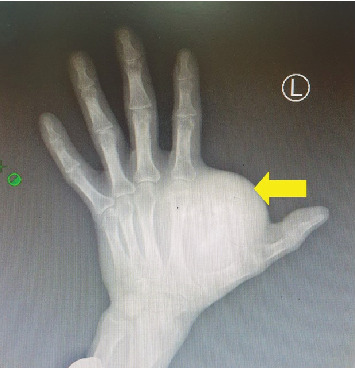
X-ray left hand showing a soft tissue mass (yellow) between first and second metacarpals with areas of calcification with no bone erosion.

The neurovascular status of the limb was intact. The examination of other systems was unremarkable. His lab results were within normal ranges, and x-ray of the left hand showed a soft tissue lesion between the first and second metacarpals with areas of calcification (Figure 2).

**Figure 3:**
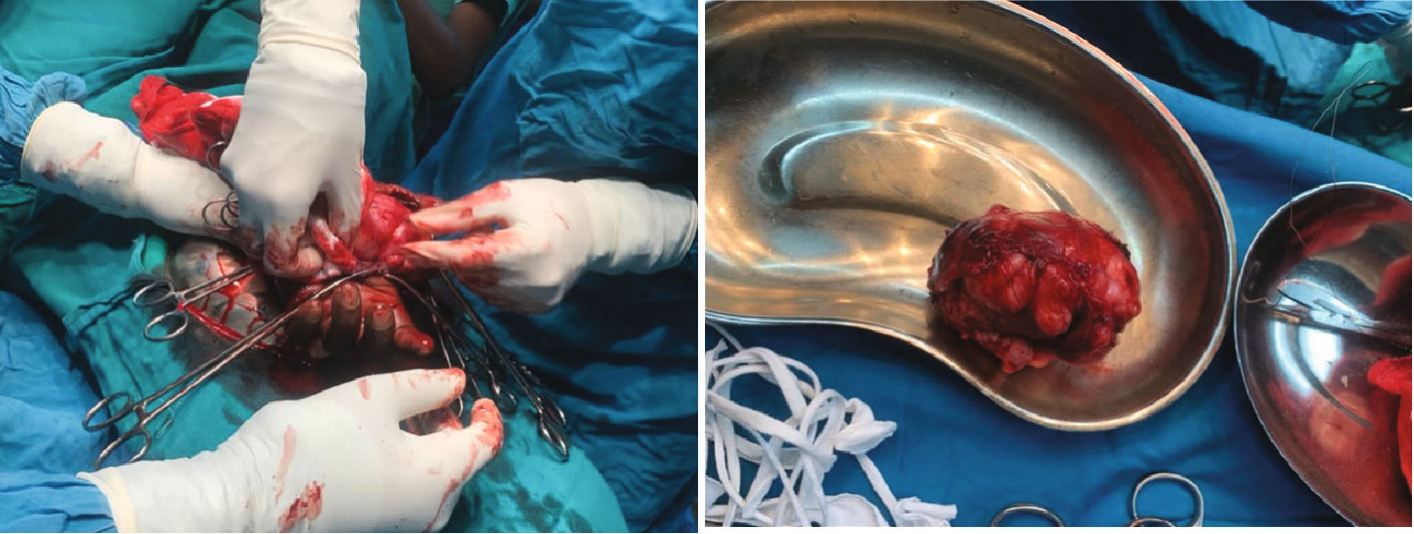
Intraoperative photographs showing spherical sarcoma measuring > 5 cm in greatest diameter.

After thorough counseling and obtaining informed consent, the patient was scheduled for a wide local excision of the mass under regional anesthesia. A thenar crease incision was made on the palm, with a slight extension toward the dorsum to provide better access to the posterior aspect. The entire firm mass, measuring 12 × 14 cm, was excised in one piece. The mass was spherical in shape (Figure 3) and was located within the thenar space, attached to the extensor tendon of the index finger and the abductor muscle groups. It weighed approximately 60 g and was sent for histopathological analysis.

**Figure 4:**
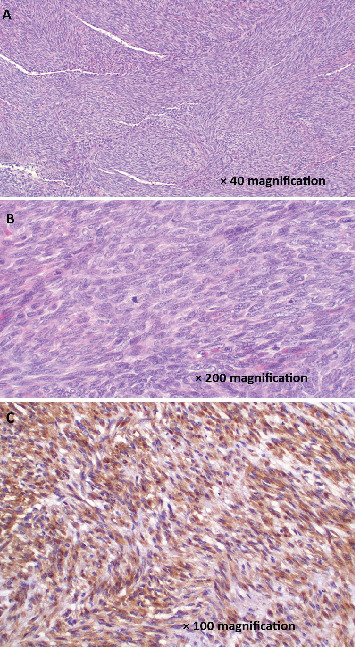
(A) Histopathology of monophasic synovial sarcoma composed of fascicles of bland spindle cells with sparse cytoplasm and relatively uniform, ovoid hyperchromatic nuclei and inconspicuous nucleoli. Neither intervening stroma nor necrosis is identified (H&E staining). (B) The tumor cells are spindled with blunt-ended nuclei, variable pleomorphism, and occasional sparse mitoses (H&E staining). (C) Photomicroscope of the tumor cells demonstrating immunostaining with CD99 (IHC staining).

Histopathology confirmed the diagnosis of synovial sarcoma (Figure 4).

**Figure 5:**
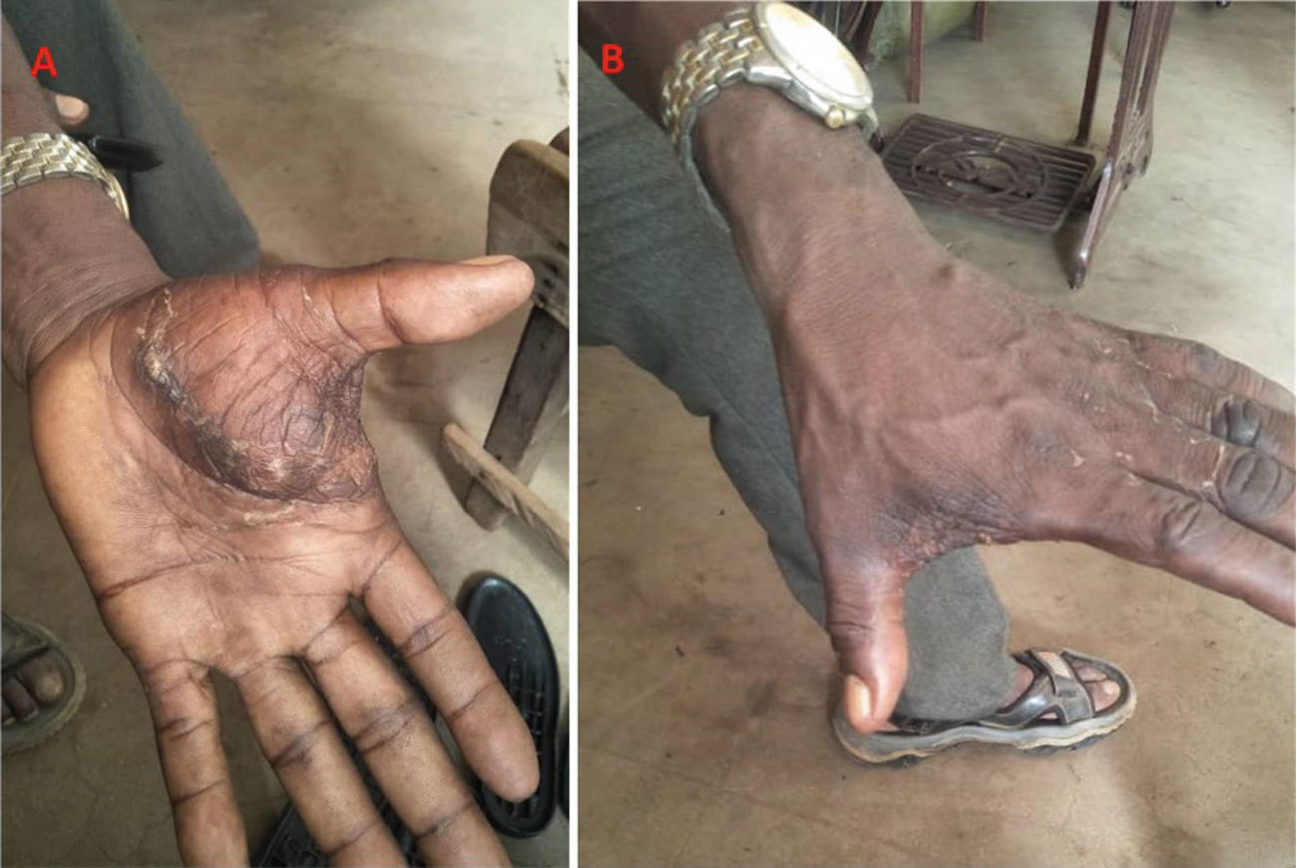
(A, B) Follow-up clinical photograph showing healed surgical scar with no features of recurrence; hand function maintained.

Postoperatively, the patient was managed in the general surgical ward for 4 days, receiving oral analgesics and prophylactic antibiotics according to local hospital protocols. Upon discharge, the patient was reviewed at the surgical outpatient unit 8 weeks later. At that time, the surgical incision had healed well, with no signs of local recurrence, and the hand function was optimal (Figure 5). The patient was subsequently referred to the oncology department for regular follow-up and further management.

## 3. Discussion

Synovial sarcomas are rare malignant soft tissue tumors that account for 5%–10% of all soft tissue sarcomas, with a unique combination of mesenchymal and epithelial differentiation. These tumors typically affect adolescents and young adults, with a slight male predominance, and are most commonly found in the extremities [4]. Occurrence in individuals over 50 is rare, making our patient's case, a 57-year-old African male, particularly unusual. Clinically, synovial sarcomas tend to grow slowly and are often painless, which can lead to misdiagnosis as benign conditions such as ganglion cysts or glomus tumors, especially when located near the wrist. Some studies show that it can take 2–5 years to diagnose from first symptoms [5, 6].

The radiological features of synovial sarcomas are often varied and subtle, making them challenging to diagnose. These features are not pathognomonic, contributing to delays in diagnosis or misdiagnosis. In some cases, they may present with traumatic pain, further complicating the clinical picture. As a result, synovial sarcomas are frequently mistaken for other conditions such as myositis, hematomas, tendinitis, bursitis, or abscesses, which can delay appropriate treatment [7]. SS are known for their high metastatic potential, and their primary histologic subtypes include monophasic, biphasic, and poorly differentiated forms. The monophasic subtype is most common and composed only of spindle cells, as the case presented herein, and the biphasic subtype contains both spindle cells and epithelial cells. The poorly differentiated subtype is composed of cells resembling those found in small round blue cell tumors [8].

Despite their name, these tumors are typically deep-seated and extra-articular, as seen in our index case. A distinctive feature of synovial sarcomas is the presence of a chromosomal translocation, t(X; 18)(p11; q11), resulting in the SS18-SSX1 or SS18-SSX2 fusion genes. Detection of these fusion genes through cytogenetic or molecular genetic techniques serves as an accurate diagnostic tool [9]. Unfortunately, due to resource limitations, this testing was not performed in our case.

Additional markers—such as TLE1, EMA, BCL2, and cytokeratins—would have strengthened the pathological confirmation but were unavailable due to resource constraints. As highlighted by Righi et al., the diagnosis of synovial sarcoma is ideally based on a combination of morphological and immunohistochemical features, with molecular confirmation of the SS18-SSX fusion considered desirable, particularly for unusual presentations or small biopsies. Recently, novel SS18::SSX and SSX C-terminus–specific antibodies have shown high sensitivity and specificity for synovial sarcoma, offering the potential for immunohistochemistry to replace molecular genetics in most cases. Unfortunately, neither molecular confirmation nor these newer antibodies were available in our setting, which should be noted as a diagnostic limitation [10].

Synovial sarcoma is morphologically diverse and is typically classified into three main histological subtypes: monophasic, biphasic, and poorly differentiated forms. From an immunohistochemical perspective, traditional markers such as EMA, AE1/AE3, BCL2, and CD99 remain widely used, while TLE1 has emerged as a particularly sensitive marker in supporting the diagnosis. Recent studies have suggested that a broader panel—including TLE1, SOX2, PAX7, INI1, and NKX3.1—may serve as valuable ancillary tools to improve diagnostic accuracy. Fluorescence in situ hybridization (FISH) can further assist in confirmation; for example, atypical SS18 break-apart patterns, including complete loss of the green signal, have been described, along with the identification of novel fusion sequences in rare cases. Alterations involving the EWSR1 gene have also been reported in a small subset of synovial sarcomas [11].

The primary treatment for SS remains surgical resection, often accompanied by radiotherapy. While synovial sarcomas are considered chemo-sensitive, the overall efficacy of chemotherapy is still under debate. Chemotherapy is typically not used in those tumors more than 5 cm. Key prognostic factors that influence survival include patient age, tumor stage, size, site (extremities vs. trunk), and the occurrence of local recurrence. In our case, the patient's age, tumor size, and coexisting HIV infection place him in a higher risk category for poor outcomes. However, the 1-year follow-up was promising, with no metastasis detected, and the tumor was successfully resected despite its chronicity [12, 13].

We acknowledge several limitations in this case. Firstly, the relatively short 1-year follow-up period limits the ability to fully assess long-term outcomes, as late recurrences and metastases are possible. The patient was counseled on red-flag symptoms—such as rapid swelling, pain, or functional impairment—that should prompt urgent reassessment, although yearly follow-up imaging and review would have been preferable. Secondly, molecular confirmation of the SS18-SSX fusion gene, the gold standard for diagnosis, was not performed due to resource constraints. This is important to note, as other spindle cell lesions remain in the differential diagnosis. Finally, we recommend strengthening cancer databases for solid tumors in African countries, enabling data sharing and collaborative research to better understand disease patterns, outcomes, and potential regional risk factors.

The 5-year survival rate for synovial sarcomas ranges from 70% to 80%, while the 10-year survival rate is approximately 50%. However, recurrence rates are significantly higher—up to 60% within 2 years—when adequate surgical resection with negative margins is not achieved. This underscores the importance of complete excision with clear margins. In cases where resection is not feasible, amputation may be considered to minimize the risks of both local recurrence and distant metastasis. Some authors advocate for the use of radiation therapy, either as a neoadjuvant or adjuvant treatment, to facilitate surgical resection and preserve limb function, though this remains a subject of debate [14].

## 4. Conclusion

Synovial sarcoma is an uncommon and aggressive malignancy that can closely resemble benign lesions, posing a diagnostic challenge in routine practice. This report documents the first known case from Tanzania, underscoring the need for heightened clinical vigilance to ensure timely recognition and management. Furthermore, our experience highlights the importance of establishing and strengthening integrated cancer databases across the African continent, which would serve as a vital resource to enhance epidemiological tracking, facilitate early diagnosis, and support future research initiatives.

## Data Availability

Data sharing is not applicable to this article as no new data were created or analyzed in this study.

## Nomenclature

ARV: antiretroviral
HIV: human immunodeficiency virus
SS: synovial sarcoma
